# Enhanced Antibacterial Activity of CuS-BSA/Lysozyme under Near Infrared Light Irradiation

**DOI:** 10.3390/nano11092156

**Published:** 2021-08-24

**Authors:** Abir Swaidan, Sena Ghayyem, Alexandre Barras, Ahmed Addad, Sabine Szunerits, Rabah Boukherroub

**Affiliations:** 1 University of Lille, CNRS, Centrale Lille, University Polytechnique Hauts-de-France, UMR 8520-IEMN, F-59000 Lille, France; abir.swaidan.90@gmail.com (A.S.); sghsgh.1368@gmail.com (S.G.); alexandre.barras@univ-lille.fr (A.B.); sabine.szunerits@univ-lille.fr (S.S.); 2 LEADDER, Laboratoire des Etudes Appliquées au Développement Durable et Energie Renouvelable, Lebanese University, Hadath 1417614411, Lebanon; 3 Analytical Chemistry Department, School of Chemistry, College of Science, University of Tehran, Tehran 1417935840, Iran; 4 CNRS, UMR 8207—UMET, University of Lille, F-59000 Lille, France; ahmed.addad@univ-lille.fr

**Keywords:** CuS-BSA NPs, lysozyme, drug delivery, NIR irradiation, photothermal agents, bacteria

## Abstract

The synthesis of multifunctional photothermal nanoagents for antibiotic loading and release remains a challenging task in nanomedicine. Herein, we investigated a simple, low-cost strategy for the preparation of CuS-BSA nanoparticles (NPs) loaded with a natural enzyme, lysozyme, as an antibacterial drug model under physiological conditions. The successful development of CuS-BSA NPs was confirmed by various characterization tools such as transmission electron microscopy (TEM), X-ray diffraction (XRD), Raman spectroscopy, and X-ray photoelectron spectroscopy (XPS). Lysozyme loading onto CuS-BSA NPs was evaluated by UV/vis absorption spectroscopy, Fourier-transform infrared spectroscopy (FTIR), zeta potential, and dynamic light scattering measurements. The CuS-BSA/lysozyme nanocomposite was investigated as an effective means for bacterial elimination of *B. subtilis* (Gram-positive) and *E. coli* (Gram-negative), owing to the combined photothermal heating performance of CuS-BSA and lysozyme release under 980 nm (0.7 W cm^−2^) illumination, which enhances the antibiotic action of the enzyme. Besides the photothermal properties, CuS-BSA/lysozyme nanocomposite possesses photodynamic activity induced by NIR illumination, which further improves its bacterial killing efficiency. The biocompatibility of CuS-BSA and CuS-BSA/Lysozyme was elicited in vitro on HeLa and U-87 MG cancer cell lines, and immortalized human hepatocyte (IHH) cell line. Considering these advantages, CuS-BSA NPs can be used as a suitable drug carrier and hold promise to overcome the limitations of traditional antibiotic therapy.

## 1. Introduction

Infection by bacteria is a serious concern to human health. Currently, the use of antibiotics remains the main treatment of infections caused by bacteria. However, antibiotics’ excessive and improper use have promoted the development of multidrug-resistant bacteria, which cause long-term adverse side effects [1].

Compared with synthetic antibiotics [2], natural antibacterial agents, such as lysozyme from chicken egg white, have gained much more attention thanks to their biocompatibility and biodegradability [3]. Lysozyme was first discovered in 1922 by Alexander Fleming [4]. It was then considered as a glycoside hydrolase enzyme whose antimicrobial properties were recognized by Pavel Nikolaevich Lashchenkov in 1909 [5]. Lysozyme was also reported as an antibacterial enzyme against food poisoning bacteria [6]. Besides its antimicrobial action, several additional activities have been attributed to this hen egg white, including tumor cell lysis and antiviral defense, among others [7]. The wide spectrum of lysozyme makes it a promising platform in pharmaceuticals, food preservation, and biomedical applications [8]. Lysozyme is well-known in inhibiting Gram-positive bacterial strains by damaging the peptidoglycan in bacterial cell wall through hydrolysis the 1,4-β-linkage between *N*-acetyl-muramic acid and *N*-acetyl-d-glucosamine [9,10]. In contrast, lysozyme has been shown to be less effective towards Gram-negative strains owing to the presence of a lipopolysaccharide (LPS) protective layer on the bacteria cell wall [11]. Despite the myriad applications of this antibiotic, its low stability and weak binding affinity to bacterial membrane, as well its denaturation ability, have restricted its research interest [12].

To address these issues, lysozyme can be prepared in microcapsule forms or integrated with other antibiotics to enhance its activity [13,14,15]; these approaches are very promising to ensure the efficacy of the enzyme. It has been also reported that coating of lysozyme with polymers or polysaccharides such as sucrose, poly-γ-glutamic acid, and chitosan improves lysozyme quality, as well as its long-term stability and bactericidal activity [16,17,18].

Recent progress in nanotechnology and materials science have led to the expansion of nanomaterials as antibiotic carriers to improve their antimicrobial action. However, the synthesis of such nanomaterials may require tedious methodologies or involve the use of toxic chemical compounds. Therefore, the search for effective, safe, and robust multifunctional nanomaterials as alternatives to traditional antibiotic therapy becomes a growing research interest worldwide. In this regard, lysozyme immobilization or loading on nanoparticles such as carbon nanotubes [19] and ZnO nanoparticles [20] represents another strategy, which broadens the antimicrobial spectrum of lysozyme and makes it a promising releasing vehicle for bacterial treatment. Moreover, mesoporous silica nanoparticles with their tunable structure, good biocompatibility, and high loading capacity have been considered as promising drug carriers [21] for bacterial treatment [11]. These approaches have witnessed a huge development of nanomaterials for bacterial ablation.

Recent studies reported a plethora of nanomaterials with photothermal capacities as new therapeutic platforms for selective targeting of bacteria. Heat induced by photothermal nanotherapy using noble metal nanoparticles, as well as carbon-based and polymer-based nanomaterials [22], could be an efficient mechanism to kill bacterial cells [22,23]. In addition to these materials, CuS-based nanomaterials have been investigated in various fields owing to their easy synthesis and reduced toxicity, as well as specific optical, physical, and chemical properties [24], and employed as antiseptic agents [21]. The most intriguing feature of CuS-based nanomaterials is their strong near-infrared (NIR) optical absorption, making them effective photothermal agents for antimicrobial therapy by means of local hyperthermia that minimizes the therapeutic issues of conventional antibiotics [21]. Ming-Yong et al. [25] described the use of CuS-BSA NPs as an antibacterial agent under NIR irradiation. Baocheng and colleagues [26] also demonstrated the successful use of CuS-BSA NPs for in vitro photothermal treatment of bacterial infections. Moreover, Khan et al. [27] assessed the therapeutic efficacy of CuS NPs for reactive oxygen species’ generation to fight bacterial infection.

Herein, the aim of this study relies on a facile synthesis of CuS-BSA NPs platform with good dispersibility, stability, and biocompatibility for effective loading and controlled release of lysozyme under a 980 nm NIR laser, in order to extend its antibacterial action, enhance its stability, and minimize the drug resistance behavior of bacteria. This work is the first report on lysozyme loading and release from negatively charged CuS-BSA NPs for complete elimination of Gram-negative and Gram-positive bacteria.

The mechanism of bacterial fighting in this assay is based on the capability of CuS NPs to convert NIR light energy to thermal vibrations in which the bacteria can be killed by means of the combined effect of hyperthermia and the bactericidal efficiency of lysozyme. The choice of NIR light as an external physical stimulus is made on the basis of the high photothermal heating performance of CuS NPs at 980 nm, owing to the d-d band transition of Cu^2+^ ions [28,29,30], which extends light absorption to ~980 nm [31]. Under NIR irradiation, lysozyme is released from the CuS-BSA NPs and causes eradication of bacteria, while under the same conditions, the NPs show no toxic effect on mammalian cells. These fascinating highlights are encouraging for the particular treatment of infectious diseases caused by bacteria, as well as the use of multifunctional nanomaterials in drug delivery and biomedical applications, by offering outstanding benefits to overcome bacterial resistance over classical antibiotics.

## 2. Experimental Section

### 2.1. Synthesis of CuS-BSA Nanoparticles

CuS-BSA nanoparticles were synthesized via a bio-mineralization assay, according to previously reported literature with some modifications [32,33]. In a typical experiment, 70 mg of BSA was dissolved in 9.5 mL MQ-water. A blue cloudy solution was observed after adding 174 mg of Cu(Ac)_2_ under magnetic stirring. The solution pH was adjusted to 12 using an aqueous NaOH (0.5 mL, 2 mol L^−1^) solution. Then, 138 mg of Na_2_S × H_2_O was added, resulting in a brown colored solution. The reaction mixture was heated at 90 °C and kept under magnetic stirring for 12 h. CuS-BSA nanoparticles were further purified by dialysis (12–14 kDa membrane) in Milli-Q water for 24 h and stored at 4 °C for further use.

The nanoparticles (10 mg mL^−1^) were found to be very stable in aqueous media for 5 months without any noticeable aggregates’ formation. The nanoparticles’ (1 mg mL^−1^) stability and dispersibility were also examined in various solvents (Dulbecco’s modified Eagle’s medium (DMEM), dimethyl sulfoxide (DMSO), phosphate buffer saline (PBS), and bacterial culture MHB medium) for one week.

### 2.2. Lysozyme Loading onto CuS-BSA Nanoparticles

Loading of lysozyme onto CuS-BSA NPs was achieved by simple mixing of CuS-BSA (1 mg mL^−1^) aqueous solution and lysozyme (1 mg mL^−1^) in 1 mL of 0.01 mol L^−1^ PBS buffer (pH 7.4) for 24 h at 4 °C or 25 °C under moderate shaking at 150 rpm. Mixing of CuS-BSA NPs with various lysozyme concentrations under the aforementioned conditions was also performed to determine the highest loading efficiency of lysozyme. The resulting solution mixture was centrifuged (8500 rpm at 4 °C) for 10 min; the supernatant was collected after several washing steps with MQ-water and filtered twice using a Ge-Whatman filter (0.2 µm).

The amount of lysozyme loaded onto the CuS-BSA NPs was determined using UV–vis measurements at 279 nm and calculated using Equation (1). Lysozyme features an absorption maximum at 279 nm, which mostly scales linearly with its concentration in solution.

(1) $${\left[\mathrm{Lysozyme}\right]}_{\mathrm{loaded}\;}={\left[\mathrm{Lysozyme}\right]}_{\mathrm{initial}\;}-{\left[\mathrm{Lysozyme}\right]}_{\mathrm{supernatant}\;}\left(\mu g/\mathrm{mL}\right)$$

where [Lysozyme]_loaded_ is the concentration of lysozyme loaded onto CuS-BSA, [Lysozyme]_initial_ corresponds to the initial concentration of lysozyme in solution, and [Lysozyme]_supernatant_ represents the concentration of free lysozyme in the supernatant.

A calibration curve, generated at 279 nm using lysozyme solutions of different concentrations, was used to determine the amount of lysozyme loaded onto CuS-BSA NPs.

To evaluate the loading efficiency, the content of lysozyme in the supernatant was calculated by UV–vis measurements at 279 nm using the following equation (Equation (2)):

(2) $$\mathrm{LE}\;\%=\frac{I\left(\mathrm{lysozyme}\right)-S\left(\mathrm{lysozyme}\right)}{I\left(\mathrm{lysozyme}\right)}\times 100\;$$

where LE is the loading efficiency of lysozyme and I_lysozyme_ and S_lysozyme_ are the contents of initial and free lysozyme in the supernatant, respectively.

The pellet that corresponds to the CuS-BSA/Lysozyme nanocomposite was re-dispersed in 1 mL of fresh PBS solution for subsequent lysozyme release.

### 2.3. NIR Light Induced Photothermal Heating of CuS-BSA Nanoparticles

The photothermal heating performance of different CuS-BSA and CuS-BSA/lysozyme (0–200 µg mL^−1^) were examined in 0.01 mol L^−1^ PBS solution in a total volume of 1 mL in 24-well polystyrene plates under NIR irradiation. A 980 nm continuous wave laser (Gbox model, Fournier Medical Solution, Lille, France) at different power densities (0.7 or 1.0 Wcm^−2^) was used. The irradiation time was 3 min. Thermal images were acquired with an infrared camera (Thermovision A40, Wilsonville, OR, USA) and treated by ThermaCam Researcher Pro 2.9 software.

### 2.4. In Vitro Photothermal and Passive Release of Lysozyme

Release experiments were investigated by mixing 0.1 mL of CuS-BSA/lysozyme (1 mg mL^−1^) with 0.9 mL of 0.01 mol L^−1^ PBS, and subjecting the mixture to 980 nm illumination (power density = 0.7 or 1.0 W cm^−2^) for 3 min. After irradiation, the mixture was centrifuged (5 min at 13,500 rpm), the supernatant was collected and filtered over a Ge-Whatman filter (0.2 µm), and its absorbance at 279 nm was determined by UV–vis spectrometry.

We also tested the passive release of lysozyme from CuS-BSA in 1 mL of PBS at different temperatures (4, 25, and 37 °C) and for various time intervals (4, 6, and 12 h).

### 2.5. Cell Culture and Cytotoxicity Studies

The cytotoxicity of CuS-BSA and CuS-BSA/Lysozyme was examined on two cancer cell lines, HeLa (human cervical adenocarcinoma) and U-87 MG (human glioblastoma astrocytoma) (ECACC 93021013 and ECACC 89081402, respectively, Sigma-Aldrich, St. Quentin Fallavier, France), and immortalized human hepatocyte (IHH) cell lines, without and with NIR irradiation at different laser power densities.

Cells were cultured in DMEM or William’s E medium containing 10% FBS (fetal bovine serum) and 1% penicillin-streptomycin at 37 °C under 5% CO_2_. Thereafter, cells (100 µL) were plated into 96-well plates at 1 × 10^4^ cells/well in DMEM or William’s E medium and incubated for 24 h before assay. The cells were then exposed to CuS-BSA and CuS-BSA/lysozyme (0–200 μg mL^−1^) for another 24 h. The old medium was removed and replaced with a fresh medium.

Prior to laser treatment, cells were incubated with CuS-BSA and CuS-BSA/lysozyme for 4 h at 37 °C and then irradiated with a 980 nm (1.0 W cm^−2^) continuous mode laser for 3 min and incubated for an additional 20 h.

The cell viability was assessed using the resazurin cell viability assay. In short, resazurin solution (11 mg mL^−1^ in PBS 1X) was diluted 1000-fold in DMEM or William’s E medium. Then, 100 µL out of this solution was introduced into each well and the plate was placed in an incubator for 3 h. Fluorescence emission of each well was recorded at 593 nm (20 nm bandwidth) at an excitation wavelength of 554 nm (18 nm bandwidth) using a Cytation™ 5 Cell Imaging Multi-Mode Reader (BioTek Instruments SAS, Colmar, France). All conditions were performed in triplicates, and the mean fluorescence value of non-exposed cells was taken as 100% cellular viability.

### 2.6. Bacterial Culture and Enzyme Activity Assay

Gram-positive *B. subtilis* 168 and Gram-negative *E. coli* K12 bacteria were selected to evaluate the antibiotic activity of the photothermally released lysozyme from CuS-BSA NPs. In a typical procedure, a single isolated colony of *E. coli* K12 or *B. subtilis* from LB or MHB agar plates was inoculated in 8 mL of LB or MHB medium. After incubation at 37 °C for 12 h under moderate shaking (150 rpm), the pre-culture was diluted by 50–fold and incubated for another 4–6 h to reach OD_600_ 0.5–1.0 [34]. Here, 1 mL of nutrient medium (LB or MHB) was used as the blank for calibrating the spectrophotometer. The bacterial culture was diluted to 10^6^ CFU mL^−1^ in the desired medium. Then, 100 µL out of the diluted bacterial culture was mixed with 100 µL of lysozyme, CuS-BSA, or CuS-BSA/lysozyme at various concentrations (0–200 µg mL^−1^ in PBS) in 96-well plates, and incubated at 37 °C for 18–24 h. Bacterial culture in the absence of NPs was considered as a growth control (GC) and treated as above.

Prior to photothermal ablation of bacterial strains, 0.5 mL of the bacterial culture (10^6^ CFU mL^−1^) was incubated at 37 °C with 0.5 mL of CuS-BSA or CuS-BSA/lysozyme (0–200 µg mL^−1^ in PBS) in 24-well plates for 30 min before irradiation. As controls, 0.5 mL of bacterial culture mixed with 0.5 mL lysozyme (0–200 µg mL^−1^) or sterile 0.01 mol L^−1^ PBS was subjected to 980 nm NIR-laser (0.7 or 1.0 W cm^−2^) for 3 min.

The turbidimetric and micro-dilution techniques were used to assess the minimal inhibitory concentration (MIC) and minimal bactericidal concentration (MBC) of the nanoparticles, respectively. Thus, a plating method was applied to count the number of viable bacterial cells, through diluting the bacterial/NPs solution by 10-fold in PBS in 96-well plates, followed by depositing 10 µL out of the diluted solution onto an LB or MHB agar plate and incubation at 37 °C for 18–24 h. The number of visual colonies was counted and calculated using Equation (3), which allowed the determination of the final concentrations of *E. coli* and *B. subtilis* strains. The bacterial growth rates were measured using a microplate reader using the absorbance at 600 nm (Cytation™ 5 Cell Imaging Multi-Mode Reader, BioTek Instruments SAS, Colmar, France).

(3) $$\mathrm{Number}\;\mathrm{of}\;\mathrm{Colonies}/\mathrm{mL}\;=\frac{\mathrm{Number}\;\mathrm{of}\;\mathrm{Colonies}}{\mathrm{Volume}\;\mathrm{plated}\;\times \;\mathrm{total}\;\mathrm{dilution}\;\mathrm{used}}\;\left(\mathrm{CFU}/\mathrm{mL}\right)$$

### 2.7. Photodynamic Properties of CuS-BSA Nanoparticles

UV–vis absorption spectroscopy was used to estimate the singlet oxygen (^1^O_2_) generation by CuS-BSA NPs under NIR illumination, through monitoring the decrease in absorbance intensity at 415 nm of 1,3-diphenylisobenzofuran (DPBF) [35]. DPBF is well-known as a singlet oxygen trapping agent that forms a bleached adduct when it reacts with ^1^O_2_. Typically, 0.1 mL of DPBF (0.8 mmol L^−1^ in ethanol) was mixed with 0.1 mL of (a) 1 mg mL^−1^ CuS-BSA or (b) 1 mg mL^−1^ CuS-BSA/lysozyme, and the total volume was adjusted to 1.0 mL using MQ-water. The samples were then irradiated at 980 nm (0.7 W cm^−2^) for different times, and the decrease in absorbance at 415 nm was then recorded. As a control, DPBF absorbance intensity was also recorded after irradiation under the same conditions mentioned above.

## 3. Results and Discussion

### 3.1. Preparation and Characterization of CuS-BSA Nanoparticles

The CuS-BSA nanoparticles were synthesized using a simple method, and found to be highly stable in solution for 5 months. The stability of the synthesized nanoparticles was further examined by their dispersion in different solvents (e.g., DMEM, DMSO, PBS, and bacterial nutrient medium). CuS-BSA NPs are highly dispersible in various solutions (Figure S1) over one week without any apparent aggregates.

**Figure 1:**
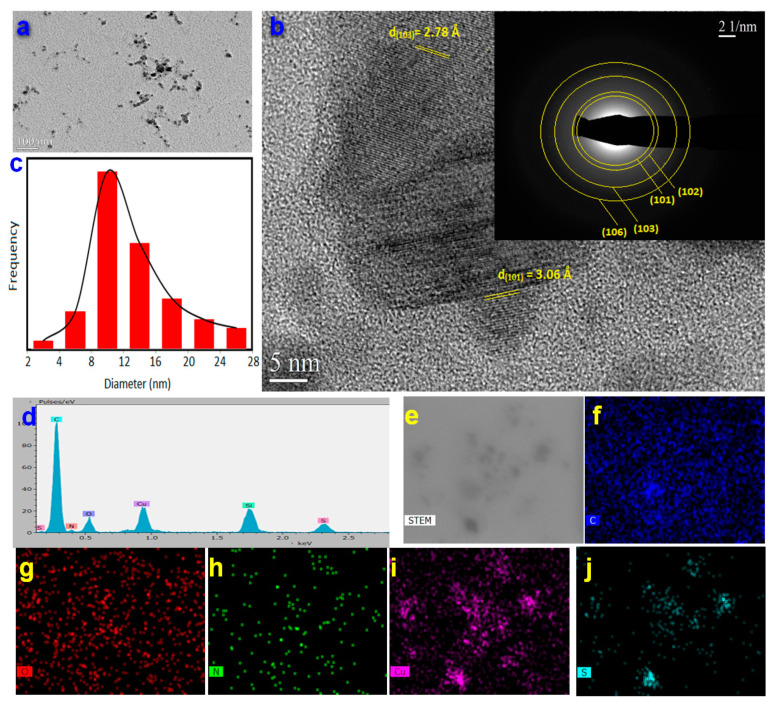
(**a**) TEM image; (**b**) HRTEM image and SAED pattern (inset **b**); (**c**) particle size distribution; (**d**) EDX spectrum; (**e**) DF-STEM image; and elemental mapping for (**f**) carbon, (**g**) oxygen, (**h**) nitrogen, (**i**) copper, and (**j**) sulfur of CuS-BSA nanoparticles.

The successful preparation of the nanoparticles was confirmed by various characterization techniques. The morphology of CuS-BSA nanoparticles was studied by transmission electron microscopy (TEM) analysis (Figure 1). TEM images revealed CuS-BSA nanoparticles of 9.7 ± 0.6 nm average size (the size of 100 NPs was determined using Image J software) (Figure 1a,c), with lattice fringes of 0.278 and 0.306 nm corresponding to the (103) and (102) planes of CuS, respectively. As depicted in the SAED pattern (inset of Figure 1b), CuS-BSA NPs are of crystalline nature with obvious (101), (102), (103), and (106) diffraction planes. Elemental mapping revealed the presence of C, N, O, Cu, and S elements in the CuS-BSA nanoparticles.

**Figure 2:**
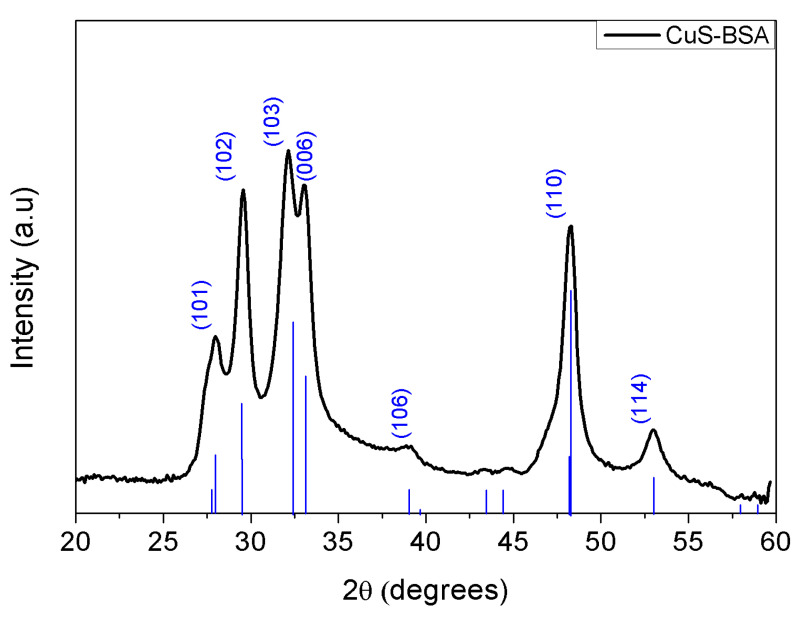
X-ray diffraction (XRD) plot of CuS-BSA NPs.

The structure of CuS-BSA NPs was investigated by X-ray diffraction (XRD). As seen in Figure 2, the diffraction peaks of CuS-BSA match well the JCPDS No.06-0464 of covellite CuS (space group 194, P63/mmc). The diffraction peaks at *2*θ values of 27.96°, 29.56°, 32.13°, 33.02°, 39.12°, 48.29°, and 53.00° [36,37,38,39] corresponding to the (101), (102), (103), (006), (106), (110), and (114) diffraction planes, respectively, with d-spacing values of 3.18, 3.01, 2.78, 2.71, 2.30, 1.88, and 1.72 Å, respectively, are characteristic of CuS NPs and consistent with the TEM data analysis.

The average crystallite size was calculated by the Debye–Scherrer equation (Equation (4)) [40]:

(4) $$D=\frac{k\lambda }{\beta \;cos\theta }\;$$

where D corresponds to the crystallite size, *k* is the geometrical factor (0.9 for spherical crystals), *λ* represents the wavelength of incident radiation (1.54), *β* illustrates the full width at half maximum (FWHM, radians), and *θ* is the Bragg angle (radians). By utilizing the FWHM of the (110) diffraction plane, an average crystallite size of 9.59 nm was calculated.

The Raman spectrum of CuS-BSA nanoparticles (Figure S2) displays a pronounced peak at 480 cm^−1^ related to the stretching mode of the Cu-S bond [41,42] and a weak peak at ~270 cm^−1^ attributed to the S–S vibration [43]. The additional peaks correspond to amide I (1500–1800 cm^−1^), which arises from C=O stretching; amide III (1380 cm^−1^), assigned to C–N stretching and N–H bending vibration; and amide A (3520 cm^−1^), attributable to N–H stretching in BSA, in addition to OH, C–O, and Si–OH stretching modes at 2890 and 981 cm^−1^, respectively [44]. The peak at 520 cm^−1^ corresponds to Si substrate [45].

Fourier-transform infrared spectroscopy (FTIR) was acquired to assess the formation of CuS-BSA nanoparticles and investigate the possible interaction with lysozyme. The FTIR spectrum of pure lysozyme (Figure S3) comprises bands at 1236, 1537, 1654, and 2961 cm^−1^, as well as a broad band at 3061 cm^−1^, related to amide III, amide II (C–N stretching or N–H bending vibrations), amide I (C=O), amide A (–NH), and OH stretching [17,46], respectively. The FTIR plot of CuS-BSA features stretching vibrations ascribed to –OH (3452 cm^−1^) [47,48], carbonyl group (1643 cm^−1^), and amide II (C–N stretching) from BSA. In the FTIR curve of CuS-BSA/lysozyme, the peak intensities at 1537 and 2961 cm^−1^ related to amide II (N–H bending) and amide A (–NH) showed an obvious decrease, which indicates the interaction of lysozyme with CuS-BSA.

**Figure 3:**
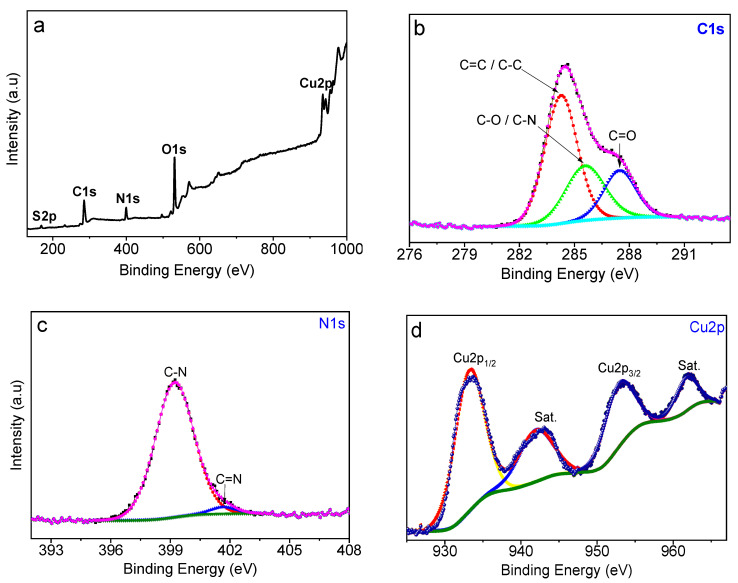
(**a**) Wide scan and core-level XPS plots of (**b**) C_1s_, (**c**), N_1s_, and (**d**) Cu_2p_ of CuS-BSA NPs.

X-ray photoelectron spectroscopy (XPS) was acquired to examine the chemical composition, purity, and valence states of CuS-BSA NPs. The XPS wide scan of CuS-BSA (Figure 3a) indicates the presence of C_1s_ (284.5 eV), N_1s_ (399.2 eV), O_1s_ (531.2 eV), Cu_2p_ (933 and 952.8 eV), and S_2p_ (162.3 eV), with atomic concentrations of 50.48, 10.70, 30.22, 6.75, and 1.84 at.%, respectively, in good agreement with the material’s chemical composition.

The core-level XPS plot of C_1s_ (Figure 3b) can be deconvoluted into several sets at 284.8, 285.5, and 287.6 eV, assigned to C–C/C=C, C–N/C–O, and C=O groups, respectively [49,50]. The O_1s_ high resolution plot (Figure S4a) could be fitted with one component at 531.2 eV ascribed to oxygen in C=O and O–H groups. The N_1s_ plot (Figure 3c) can be deconvoluted into C–N (399.2 eV) and C=N (401.6 eV) of BSA [51,52]. The Cu_2p_ core-level spectrum (Figure 3d) illustrates the deconvoluted peaks at 934.0 and 952.8 eV, corresponding to Cu_2p3/2_ and Cu_2p1/2_, respectively. The characteristic satellite peaks at ~942.8 and 962.0 eV indicate the presence of Cu^2+^ oxidation state of CuS [53]. The binding energy peaks at 162.4 and 163.4 eV in the S_2p_ spectrum (Figure S4b) correspond to S_2p3/2_ and S_2p1/2_ of CuS, respectively. The bands at 168.7 and 170.0 eV related to S_2p3/2_ and S_2p1/2_, respectively, indicate the presence of oxidized sulfur [54].

To qualitatively confirm the loading of lysozyme onto CuS-BSA, the UV–vis absorption of CuS-BSA/lysozyme was acquired along with those of CuS-BSA and free lysozyme, for comparison (Figure S5). The UV–vis absorption spectrum of CuS-BSA exhibits absorption features in the visible region (400–600 nm) with an enhanced absorption tail in the NIR region, which are characteristics of CuS NPs [55]. Meanwhile, lysozyme displays a typical absorption band at 279 nm [17]. In the UV–vis absorption plot of CuS-BSA/lysozyme, the presence of an absorption feature at 279 nm and an NIR absorption tail are the clear fingerprints of the successful loading of lysozyme onto CuS-BSA NPs.

Lysozyme with an isoelectric point of 11.35 [16] is considered to be positively charged at physiological pH. Thus, the use of negatively charged CuS-BSA NPs can enhance the adsorption of lysozyme through electrostatic interactions. Thereby, the successful loading of lysozyme on CuS-BSA NPs was also confirmed through hydrodynamic size and surface charge measurements using DLS and zeta potential, respectively. The average hydrodynamic diameter of bare CuS-BSA NPs was found to be 122 ± 8.8 nm (Figure S6), with a surface charge of −18.6 ± 2.4 mV (Figure S7), owing to the presence of hydroxyl and carboxyl groups in BSA [26]. After lysozyme loading, an average size of 158.2 nm and a surface charge of +7.13 ± 3.28 mV were recorded, indicating the successful adsorption of positively charged lysozyme onto a negatively charged CuS-BSA surface; the positive charge of CuS-BSA/lysozyme is favorable for attachment to the negatively charged bacterial surfaces [56]. The DLS size of the NPs is higher than that determined by TEM analysis owing to the hydration shell formed by water molecules on the nanoparticle’s surface [57,58].

### 3.2. Lysozyme Loading Efficiency

Lysozyme loading onto CuS-BSA was established by UV–vis absorption at 279 nm using an established calibration plot (Figure S8) under physiological neutral conditions in 0.01 mol L^−1^ PBS. A maximum loading of 0.76 mg mL^−1^ of lysozyme onto 1 mg mL^−1^ CuS-BSA at 4 °C was achieved. The loading efficiency (LE %) significantly increased with the increase of lysozyme concentration from 0.1 (LE = 43%) to 1 mg mL^−1^ (LE = 76%) (Figure S9). The loading efficiency was also tested at RT. As can be seen in Figure S10, 63.4% of lysozyme was loaded onto 1 mg mL^−1^ CuS-BSA NPs.

The long-term stability of the CuS-BSA/Lysozyme was investigated by studying the antibacterial action of the same NPs against *E. coli* after being stored for 2 months at 4 °C. The antibacterial activity was comparable to that of fresh nanoparticles, suggesting the high stability of the nanoparticles.

### 3.3. Photothermal Heating Performance

**Figure 4:**
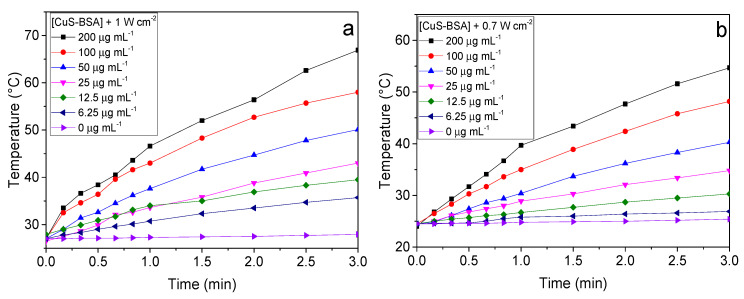

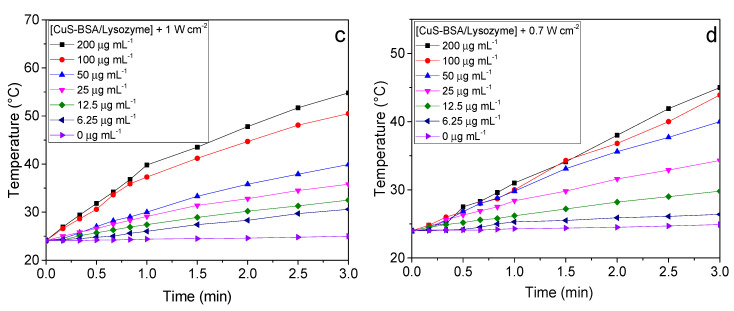
Photothermal heating curves of (**a**,**b**) CuS-BSA and (**c**,**d**) CuS-BSA/lysozyme at various concentrations in 0.01 mol L^−1^ PBS under NIR illumination (980 nm) under a continuous wave laser irradiation at different power densities for 3 min.

CuS-BSA NPs are recognized as a strong absorption photothermal agent in the NIR region. Therefore, the photothermal behavior of CuS-BSA and CuS-BSA/lysozyme at various concentrations (0–200 µg mL^−1^ in PBS) was studied by recording the temperature elevation caused upon 980 nm laser irradiation at different power densities for 3 min. Under these experimental conditions, the temperature of CuS-BSA and CuS-BSA/lysozyme increased rapidly within 3 min. Figure 4 shows a remarkable increase in temperature upon increasing the sample concentration. The highest temperature reached was about 54.7 °C and 45 °C at the highest concentration of 200 µg mL^−1^ for CuS-BSA (Figure 4b) and CuS-BSA/Lysozyme (Figure 4d), respectively, at 0.7 W cm^−2^, suggesting that the absorbed energy is rapidly converted to higher thermal energy. The heating rate was significantly enhanced when the laser power density increased to 1 W cm^−2^ to reach 66.9 °C (Figure 4a) and 54.8 °C (Figure 4c) for CuS-BSA and CuS-BSA/lysozyme, respectively.

As a control, 1 mL of PBS was subjected to irradiation under the same conditions mentioned above, where no noticeable increase in temperature was observed in the absence of CuS-BSA NPs.

Additionally, the photothermal behavior of CuS-BSA, induced by NIR-laser irradiation at 980 nm, was assessed for several ON/OFF cycles. Thus, we recorded the time-dependent temperature of CuS-BSA upon NIR irradiation for 3 min (Laser on) at 0.7 W cm^−2^, followed by cooling to room temperature when the laser is switched off for another 3 min. This cycle was recorded four times repeatedly in order to assess the photothermal stability of the CuS-BSA nanoparticles. The results in Figure S11 clearly reveal that the photothermal properties of CuS-BSA are not affected after four cycles, suggesting that the nanocarrier exhibits stable photothermal heating performance.

### 3.4. In Vitro Photothermal Lysozyme Release

As an efficient drug delivery carrier, CuS-BSA should acquire a release property. Therefore, lysozyme release from CuS-BSA was investigated by dispersing 0.1 mL of CuS BSA/lysozyme (76 µg mL^−1^ lysozyme loaded concentration) in 1 mL of PBS under NIR illumination at two different laser power densities (1 or 0.7 W cm^−2^) for 3 min. Under these conditions, 37.2 µg mL^−1^ of lysozyme was released at 0.7 W cm^−2^ (49%). Meanwhile, the release of lysozyme can be controlled by adjusting the laser power. Thus, the lysozyme release rate was slightly enhanced to reach 39.7 µg mL^−1^ (52%) within 3 min irradiation upon increasing the laser power density to 1 W cm^−2^ (Figure S12). These results suggest that lysozyme exhibits a temperature-responsive release profile, where at a higher laser power density, CuS-BSA achieved better heating performance, which enhances the detachment of lysozyme owing to the change in binding energy by NIR-mediated local heat, which reduces the interaction between lysozyme and CuS-BSA.

Furthermore, the passive release of lysozyme from CuS-BSA NPs was assessed by immersing 0.1 mL CuS BSA/lysozyme (76 µg mL^−1^ Lysozyme) in 0.01 mol L^−1^ PBS solution under moderate shaking (150 rpm) at different temperatures and incubation times. Under the aforementioned conditions, only a small fraction of lysozyme was released from CuS-BSA during the first 4 h (Figure S13). Lysozyme leakage was influenced by temperature and time of incubation, where it reaches 13.9% and 41.6% at RT and 37 °C, respectively, after 12 h. However, the passive release of lysozyme after 12 h incubation was lower than that achieved by the photothermal release under NIR irradiation for 3 min. This indicates that CuS-BSA NPs are promising therapeutic agents for drug loading and release.

### 3.5. Cellular Toxicity and Viability Studies

**Figure 5:**
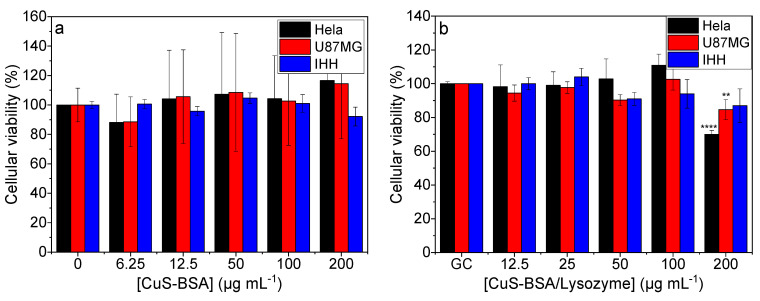
Cellular viability of (**a**) CuS-BSA and (**b**) CuS-BSA/lysozyme at various concentrations (0–200 µg mL^−1^) without irradiation on different cell lines. ** p < 0.01, **** p < 0.0001.

The cell viability studies of CuS-BSA and CuS-BSA/lysozyme were conducted on HeLa, U-87 MG, and IHH cell lines using the resazurin assay. The obtained data revealed that the cell viability remained above 80% (Figure 5a,b) even at a high concentration of CuS-BSA and CuS-BSA/lysozyme (200 μg mL^−1^), confirming the biocompatibility of the synthesized nanoparticles.

**Figure 6:**
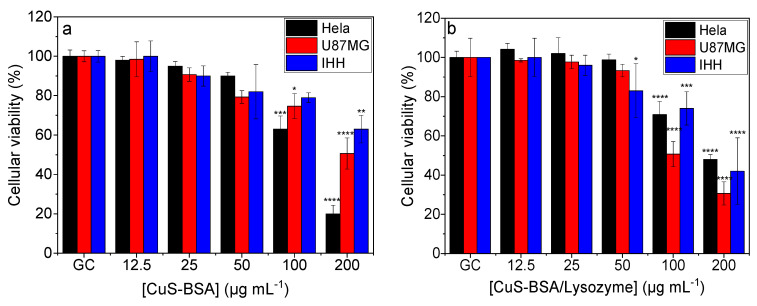
Cellular viability of (**a**) CuS-BSA and (**b**) CuS-BSA/lysozyme at various concentrations (0–200 µg mL^−1^) under 980 nm (1 W cm^−2^, 3 min) illumination on different cell lines. * p < 0.05, ** p < 0.01, *** *p* < 0.001, **** p < 0.0001.

The cytotoxicity was also assessed under NIR irradiation (1 W cm^−2^). The cells were exposed to increasing CuS-BSA and CuS-BSA/lysozyme concentrations and subjected to irradiation for 3 min. Figure 6a,b show a dose-dependent toxicity. The higher killing efficiency observed at 200 μg mL^−1^ was attributed to the strongest photothermal heating effect of CuS-BSA at this concentration (Figure 4). In a control experiment, cells were irradiated under the same conditions, where no obvious decrease in cell viability was seen.

In order to preclude the cell killing efficiency under the influence of NIR irradiation, cells were also illuminated at 1 W cm^−2^ in the absence of nanoparticles. Under these conditions, the cell viability remained at 100%, indicating that the tested cell lines are not sensitive to NIR laser irradiation, and the obtained cell killing was attributed to the photothermal heating effect of the nanoparticles (Figure 4).

Furthermore, the cell death pathway (apoptosis/necrosis) of HeLa cells was assessed using the Annexin-V-Fluorescein/PI assay [59] (Figure S14). The results revealed that laser irradiation alone did not induce necrotic cell death and the cell population under late and early apoptotic states was nearly equivalent to that of the control (cells without laser irradiation). Incubation of cells with CuS-BSA/Lysozyme at 100 and 200 µg mL^−1^ induced a higher amount of apoptotic and necrotic cells. Under the same conditions, laser irradiation revealed that the cell population was mostly necrotic. This is in agreement with the results of Zhang et al. on the cell death mechanism under photothermal conditions [60]. They found that, at temperatures between 43 and 49 °C, tumor cells’ death occurred mainly through necroptosis and apoptosis, while at a higher temperature (>49  °C), necrosis became the dominant cell death pathway. Nevertheless, after 24 h, the remaining cells attached were decreased up to 45 and 90% for 100 and 200 µg mL^−1^ incubation, respectively. Therefore, we cannot exclude a higher proportion of apoptotic cells.

### 3.6. Singlet Oxygen Generation under NIR Illumination

The singlet oxygen generation ability of CuS-BSA NPs was examined under 980 nm (0.7 W cm^−2^) irradiation through monitoring the variation of UV–vis absorbance of DPBF at 415 nm. The reaction of DPBF with singlet oxygen (^1^O_2_) generates an oxidized DPBF product (endoperoxide) [56,61], resulting in a decrease of DPBF absorbance at 415 nm. Figure S14 shows the variation of the absorbance at 415 nm (A415 nm) as a function of NIR illumination time for the CuS-BSA + DPBF, CuS-BSA/Lysozyme + DPBF, and DPBF systems. The results showed that, in the absence of CuS-BSA, there is only a slight decrease in DPBF absorbance. However, in the presence of CuS-BSA and CuS-BSA/lysozyme, there is a significant decrease in absorbance due to the generation of ^1^O_2_ under NIR irradiation, which causes DPBF oxidation. With a further increase in irradiation time, there is an enhanced bleach of DPBF, evidenced by a considerable decrease in DPBF absorption at 415 nm. This indicates that CuS-BSA could be used as a potential photodynamic therapy (PDT) agent for the generation of ^1^O_2_ under NIR illumination.

### 3.7. Antibacterial Activity

The antibacterial properties of lysozyme, CuS-BSA, and CuS-BSA/lysozyme with and without NIR illumination were assessed on *E. coli* and *B. subtilis* as model bacterial strains. Lysozyme is proven to be effective in inhibiting bacteria growth. Thus, on the basis of the optical density at 600 nm (OD_600_) (Figure S15) and plating method (Figure S16), lysozyme exhibits a decrease in the viable bacterial cells on both strains with a more pronounced toxicity to Gram-positive *B. subtilis* than *E. coli*. However, one can notice that illumination using NIR light does not induce any observable change in the number of viable cells, even at a higher laser power density (1 W cm^−2^). This indicates that lysozyme does not possess any photothermal heating capacity, and the decrease in the number of bacterial colonies with increasing concentrations of lysozyme is attributed to the bactericidal action of this antibiotic.

**Figure 7:**
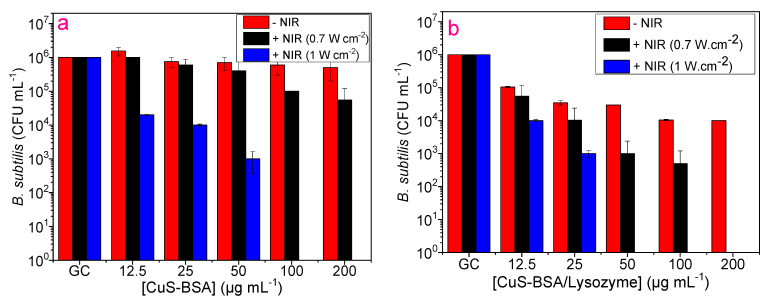
Antibacterial activity of (**a**) CuS-BSA and (**b**) CuS-BSA/lysozyme on *B. subtilis* using various concentrations with and without NIR irradiation (980 nm, 3 min).

**Figure 8:**
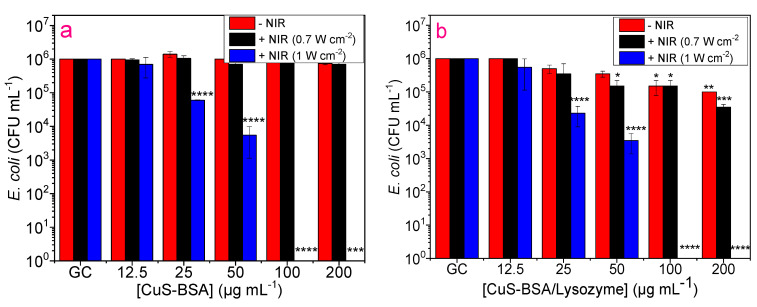
Antibacterial activity of (**a**) CuS-BSA and (**b**) CuS-BSA/lysozyme on *E. coli* using various concentrations with and without NIR irradiation (980 nm, 3 min). * p < 0.05, ** p < 0.01, *** *p* < 0.001, **** p < 0.0001.

The toxicity of CuS-BSA and CuS-BSA/lysozyme nanoparticles was also examined on *B. subtilis* and *E. coli* at various concentrations with and without NIR irradiation. As can be seen in Figure 7a, CuS-BSA displayed negligible toxicity to *B. subtilis* without irradiation. However, upon 3 min illumination (0.7 W cm^−2^), a slight toxicity was recorded at 50 µg mL^−1^ and above, which can be explained by the photothermal heating capacity at this concentration under laser treatment (47 °C). On the contrary, CuS-BSA/lysozyme (Figure 7b) resulted in a considerable reduction in bacterial viability. The illumination of *B. subtilis* at 0.7 W cm^−2^ caused complete destruction of the bacterial strain in the presence of CuS-BSA/lysozyme at 200 µg mL^−1^. The enhanced antibacterial action of CuS-BSA/lysozyme can be attributed to the strongest binding affinity to the bacterial surfaces, and the use of an NIR light source promoted the release of lysozyme from CuS-BSA and exposed the bacteria to a large amount of lysozyme [62]. This also demonstrates that the released lysozyme can still maintain good bioactivity at a higher temperature, which can effectively decompose the bacterial membrane to induce its killing efficiency. Under the aforementioned conditions, CuS-BSA NPs were less toxic to *E. coli* even under exposure to laser (0.7 W cm^−2^) irradiation. Figure 7b and Figure 8b clearly demonstrate that CuS-BSA/lysozyme possesses stronger bacterial killing efficiency to *B. subtilis* than *E. coli* owing to the difference in bacterial cell wall surfaces, as most Gram-positive bacteria secrete a protein that enhances its cell wall surface viscosity and increases the adsorption of nanoparticles [25].

Furthermore, we evaluated the influence of increasing laser power density on bacterial viability. Interestingly, both *E. coli* and *B. subtilis* were completely eradicated after 3 min exposure to laser (1 W cm^−2^) illumination using a low concentration of CuS-BSA/lysozyme of 50 µg mL^−1^ on *B. subtilis* (Figure 7b) and 100 µg mL^−1^ on *E. coli* (Figure 8b) owing to the enhanced heating effect at a higher laser power.

To rule out the effect of irradiation on bacteria without NPs, *E. coli* and *B. subtilis* were subjected to 980 nm laser treatment for 3 min at two different laser power densities, where no observable decrease in viability was detected even at a high laser power density (1 W cm^−2^). This indicates that the killing efficiency was ascribed to the combined effect of lysozyme antibacterial action and the heating effect of CuS-BSA under irradiation along with ^1^O_2_ action. Comparing both bacterial strains, *B. subtilis* was more sensitive under the same conditions, and the antibacterial action was found to be nanoparticle concentration-dependent in the 12.5 to 200 µg mL^−1^ range.

**Figure 9:**
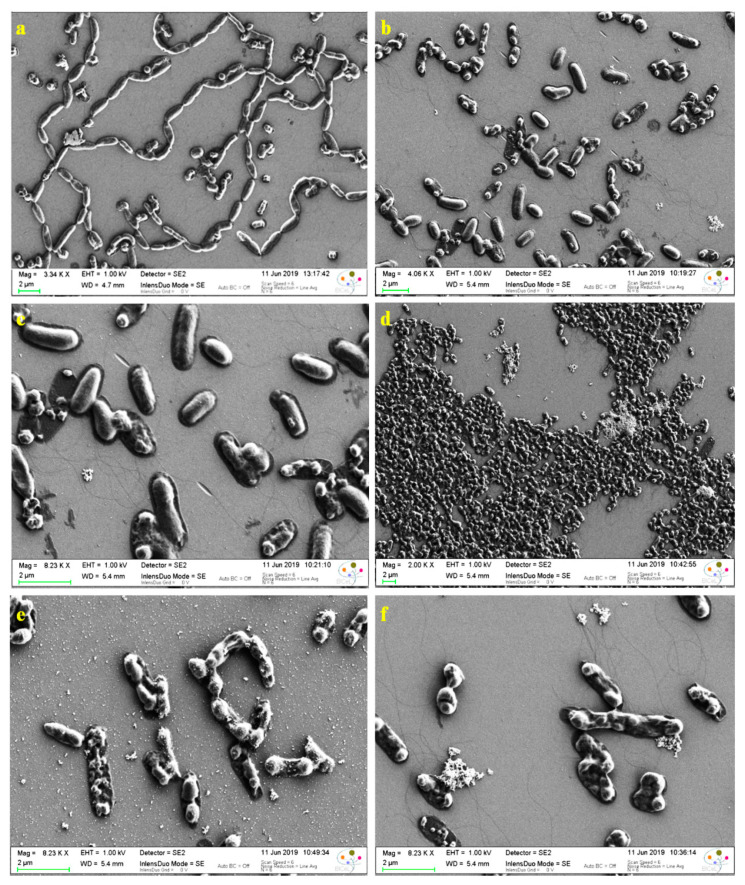
SEM images of *E. coli*: (**a**) control and incubated with CuS-BSA/lysozyme nanoparticles at a concentration of 100 μg/mL^−1^ (**b**,**c**) without and (**d**–**f**) under 980 nm (1 W cm^−2^) irradiation.

The eradication of *E. coli* was examined by SEM analysis. Figure 9a illustrates the *E. coli* bacteria, where the untreated *E. coli* shows a rod-like structure with an intact surface; however, after incubation with CuS-BSA/lysozyme, morphological changes were observed, where a few portions of the attached bacteria are decomposed, showing destroyed surfaces (Figure 9b).

The anti-bacterial activity of CuS-BSA/lysozyme (100 µg mL^−1^) against *E. coli* was investigated under NIR irradiation at 1 W cm^−2^ for 3 min. As can be seen in Figure 9d–f, lysozyme loaded onto CuS-BSA nanoparticles exhibited higher toxicity and excellent killing efficiency to *E. coli* under a 980 nm laser exposure compared with CuS-BSA/lysozyme without irradiation (Figure 9b,c), where all the bacteria are dead and exhibit disrupted surfaces, which is ascribed to the locally generated heat and lysozyme release that cause cell membrane rupture under these conditions.

**Figure 10:**
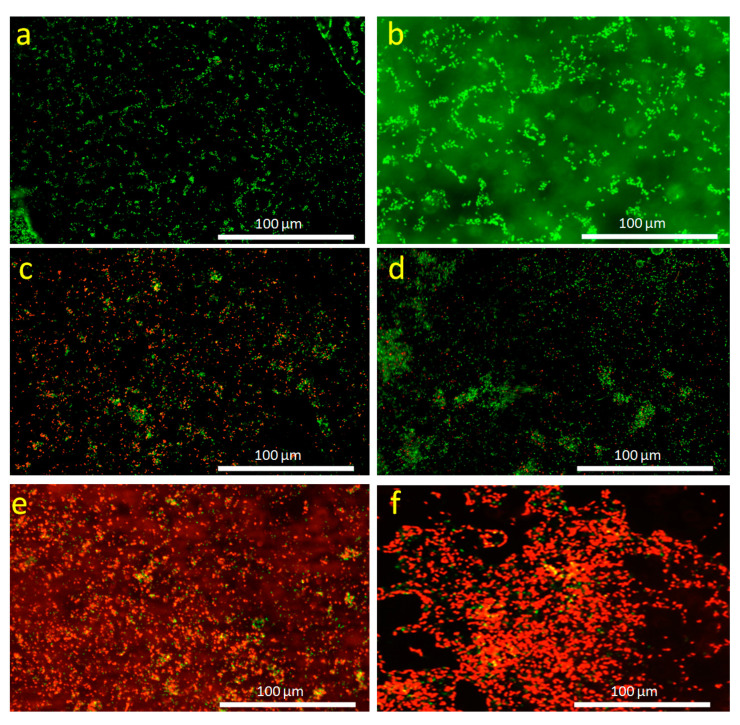
Fluorescence images of *E. coli* stained using the LIVE/DEAD Bac Light bacterial viability kit. (**a**,**b**) Control and incubated with CuS-BSA/lysozyme nanoparticles (100 μg mL^−1^) (**c**,**d**) without and (**e**,**f**) under 980 nm (1 W cm^−2^) irradiation. Note: Green color indicates viable bacteria and red color corresponds to dead bacteria.

*E. coli* eradication capability of CuS-BSA/lysozyme was also examined by fluorescence-based cell live/dead assay, in which green fluorescence is correlated to the viable live cells and the red fluorescence corresponds to the dead bacterial cells. Figure 10a,b depicts the fluorescence images of *E. coli* (control), which are dominantly composed of distinct network shapes, compromising viable cells (green color). However, upon incubation with CuS-BSA/lysozyme (Figure 10c,d), red color was observed in certain areas, indicating that some bacteria were dead. Figure 10e,f exhibits the fluorescence images of *E. coli* after incubation with CuS-BSA/lysozyme and exposure to 980 nm (1 W cm^−2^) laser illumination for 3 min, which were dominated by dead cells (red color). These results demonstrated the successful combination between photothermal heating capacity and antibiotic release of CuS-BSA nanoagents that can be applied as excellent candidates in drug delivery and PTT to achieve efficient antibacterial activity.

## 4. Conclusions

In the present work, CuS-BSA NPs were successfully synthesized via a facile method to develop a modulated photothermal and photodynamic agent as well as a drug carrier. CuS-BSA NPs were designed for NIR-light absorption, which can enhance the release of a natural enzyme, lysozyme, in vitro. Release of lysozyme from the NPs was induced by a rapid temperature increase upon 980 nm (0.7 W cm^−2^) laser irradiation within a short exposure time (3 min). CuS-BSA NPs displayed a lysozyme delivery at RT; however, a significant release was observed at a higher temperature. The photothermally-triggered released enzyme demonstrated an efficient antibacterial activity against two bacterial strains (*E. coli* and *B. subtilis*), where the results revealed that the NPs were non-toxic to bacteria at RT even at 200 µg mL^−1^. However, lysozyme release under NIR-light illumination resulted in an observable reduction in bacterial growth, with slight toxicity to mammalian cells only at higher concentrations. The excellent photothermal heating performance, high stability, efficient drug loading capacity, and release property suggest that CuS-BSA nanoparticles with multifunctional properties could be applied as a promising alternative for pharmaceutical and biomedical applications.

## Data Availability

Not applicable.

## Supplementary Materials

The following are available online at https://www.mdpi.com/article/10.3390/nano11092156/s1, Figure S1: The long-term stability of CuS-BSA NPs at a concentration of 1 mg mL^−1^ in (1) DMEM medium, (2) DMSO, (3) PBS (1X, pH 7.4), and (4) bacterial MHB nutrient medium. Figure S2: Raman spectrum of CuS-BSA NPs. The sharp peak at 520 cm^−1^ is due to crystalline silicon on which the CuS-BSA nanoparticles were deposited. Figure S3: FTIR spectra of CuS-BSA, lysozyme and CuS-BSA/Lysozyme. Figure S4: High-resolution XPS spectra of the O_1s_ (a) and S_2p_ (b) of CuS-BSA NPs. Figure S5: UV-vis absorption spectra of lysozyme (blue), CuS-BSA (black), and CuS-BSA/Lysozyme (red). Figure S6: Dynamic light scattering (DLS) curves of CuS-BSA and CuS-BSA/Lysozyme. Figure S7: Comparison of zeta potential of CuS-BSA and CuS-BSA/Lysozyme in MQ-water. Figure S8: UV-vis absorption spectra of lysozyme in PBS (0.01 mol L^−1^, pH 7.4) at various concentrations. Inset: The corresponding calibration curve. Figure S9: Lysozyme loading efficiency (LE %) by CuS-BSA in PBS (0.01 mol L^−1^, pH 7.4). Figure S10: Lysozyme loading efficiency (LE %) onto CuS-BSA in PBS (0.01 mol L^−1^, pH 7.4) at 4 °C and 25 °C. Figure S11: Temperature elevation of CuS-BSA (100 µg mL^−1^) over four laser on/off cycles under 980-NIR laser irradiation for 3 min using a power density 0.7 W cm^−2^. Figure S12: Photothermal release of lysozyme (%) from CuS-BSA NPs (0.1 mg mL^−1^) after 3 min laser irradiation at 980 nm. Experiments were done in triplicate. Figure S13: Passive release of lysozyme (%) from CuS-BSA NPs (0.1 mg mL^−1^) at various temperatures and incubation times. Figure S14: HeLa cell death mechanism under different conditions. Figure S15: Time dependent DPBF quenching experiments: (a) CuS-BSA + DPBF + NIR (0.7 W cm^−2^), (b) CuS-BSA/Lysozyme + DPBF + NIR (0.7 W cm^−2^), (c) DPBF + NIR (0.7 W cm^−2^), (d), (e) and (f) ^1^O_2_ generation efficiency upon NIR laser irradiation. Figure S16: Change of OD_600_ in the presence of various concentrations of lysozyme (µg mL^−1^) on *E. coli* and *B. subtilis.* Figure S17: Antibacterial activity of lysozyme on *B. subtilis* (a) and *E. coli* (b) using various concentrations of lysozyme without and with NIR irradiation (980 nm) at different laser power densities for 3 min.
