# Expression of Concern: Swaidan et al. Enhanced Antibacterial Activity of CuS-BSA/Lysozyme Under Near Infrared Light Irradiation. *Nanomaterials* 2021, *11*, 2156

**DOI:** 10.3390/nano16201318

**Published:** 2026-10-19

**Authors:** Nanomaterials Editorial Office

**Affiliations:** MDPI AG, Grosspeteranlage 5, 4052 Basel, Switzerland

With this notice, the *Nanomaterials* Editorial Office alerts readers to concerns regarding this article [1].

Following publication, concerns were raised regarding the integrity of certain figures included in the article [1]. In accordance with the journal’s standard procedures, the authors were contacted and asked to provide clarification and supporting documentation.

The Editorial Board reviewed the information provided by the authors. With respect to Figure 8, the authors supplied the underlying numerical data, which were evaluated and found to adequately address the concerns raised. However, the concerns relating to Figure 9 could not be fully resolved, as the authors provided evidence that could not be substantiated to represent the original underlying data associated with this figure.

In conclusion, the Editorial Board determined that the available evidence does not fully resolve the expressed concerns, but neither does it sufficiently affect the overall conclusions of the article.

In the interest of transparency and maintaining the integrity of the scholarly record, the Editorial Office is issuing this Expression of Concern to inform readers of these unresolved concerns. Based on the information currently available, no further editorial action is being taken at this time.

Should additional information become available in the future, the journal may take further action in accordance with MDPI’s policies.
