# Supplementary material for: Enhanced Antibacterial Activity of CuS-BSA/Lysozyme under Near Infrared Light Irradiation
Source: Nanomaterials (Basel). 2021 Aug 24;11(9):2156. doi: 10.3390/nano11092156 (PMC8467990; doi:10.3390/nano11092156)
Supplement: Supplementary file 1 [file nanomaterials-11-02156-s001.zip › nanomaterials-1277942-supplementary.pdf]

nanomaterials

Supporting Information

Enhanced Antibacterial Activity of CuS-BSA/Lysozyme under Near Infrared Light Irradiation

Abir Swaidan [1,2], Sena Ghayyem [1,3], Alexandre Barras [1], Ahmed Addad [4], Mohammed A. Amin [5], Sabine Szunerits [1], and Rabah Boukherroub [1,*]

[1] Univ. Lille, CNRS, Centrale Lille, Univ. Polytechnique Hauts-de-France, UMR 8520- IEMN, F-59000 Lille, France; abir.swaidan.90@gmail.com (A.S.); sghsgh.1368@gmail.com (S.G.); alexandre.barras@univ-lille.fr (A.B.); sabine.szunerits@univ-lille.fr (S.S.)
[2] LEADDER, Laboratoire des études appliquées au développement durable et énergie renouvelable, Univ. Libanaise, 1417614411 Hadath, Lebanon
[3] Analytical Chemistry Department, School of Chemistry, College of Science, Univ. of Tehran, Tehran, Iran
[4] CNRS, UMR 8207—UMET, Univ. Lille, F-59000 Lille, France; ahmed.addad@univ-lille.fr
[5] Department of Chemistry, College of Science, Taif Univ., P.O. Box 11099, Taif 21944, Saudi Arabia; mohamed@tu.edu.sa
* Correspondence: rabah.boukherroub@univ-lille.fr; Tel.: +333-62-53-17-24

Materials and Methods

Lysozyme from chicken egg white (protein ≥90%, ≥ 40,000 units/mg protein), bovine serum albumin (BSA, ≥96%), copper(II) acetate [$Cu(Ac)_2$, 98.0%], sodium sulfide hydrate ($Na_2S.{\times}H_2O$, ≥97.0%), sodium hydroxide pellets (NaOH, ≥97.0%), Mueller Hinton Agar, Muller Hinton Broth, 1,3-diphenyl isobenzofuran (DPBF, 97%) and glutaraldehyde solution (Grade II, 25% in water) were purchased from Sigma-Aldrich. LB (Luria-Bertani) Broth Miller and LB Agar Miller were purchased from Fisher Bioreagents. Propidium iodide (PI, 668.4 g/mole) and SYTO 9 nucleic acid stain (3.34 mM) (BacLight stains), sterile PBS (1X, pH 7.4) and ethanol (95% v/v) were obtained from Fisher Scientific.

Sterile Milli-Q (MQ) water was used throughout the whole procedure.

Characterization

The UV-vis absorbance spectra of the synthesized nanoparticles (NPs) were recorded in the 200-800 nm wavelength range using a Perkin Elmer Lambda UV/vis 950 spectrophotometer with an optical path of 10 mm in a 1 cm quartz cuvette.

Hydrodynamic size and surface charge of the CuS-BSA and CuS-BSA/Lysozyme NPs were determined using dynamic light scattering (Malvern Zetasizer, NanoZS) at 25 °C. Solutions were freshly prepared before analysis, and the reported data are means for three independent measurements.

Morphology and particle size of CuS-BSA NPs were investigated by transmission electron microscopy (TEM) and high resolution TEM (HRTEM) using a FEI TECNAI G2-20 operated at an accelerating voltage of 200 kV with a resolution of 1.9 Å and equipped with EDS detector. Analysis was carried out by depositing ~10 µL of CuS-BSA aqueous solution (0.1 mg/mL) on a carbon 200 mesh grid and left to dry at room temperature.

Raman spectroscopy measurements were carried out by depositing 30 µL of an aqueous CuS-BSA NPs (0.1 mg/mL) on a crystalline silicon wafer and dried at room temperature; the silicon wafer was previously cleaned by ultrasonication in acetone, ethanol, and water for 10 min each. Analysis was performed on a LabRam HR Micro-Raman system (Horiba Jobin Yvon) using a 473-nm laser diode as an excitation source. Visible light is focused by a 100× objective. The scattered light was collected by the same objective in backscattering configuration, dispersed by a 300 mm focal length monochromator and detected by a CCD camera.

The crystallinity of the synthesized nanoparticles was assessed on a Xeuss 2.0 SAXS/WAXS system (Xenocs) operating under vacuum with a GeniX3D microsource (λ = 1.54189 Å) at a generator voltage of 50 kV and a current of 0.6 mA with a 2D Pilatus 3R 200K detector.

X-ray photoelectron spectroscopy (XPS) experiments were performed on a PHI 5000 VersaProbe-Scanning ESCA Microprobe (ULVAC-PHI, Japan/USA) instrument at a base pressure below 5×10^{-9} mbar. Monochromatic AlK_α radiation was used and the X-ray beam, focused to a diameter of 100 µm, was scanned on a 250 × 250 µm surface, at an operating power of 25 W (15 kV). Photoelectron survey spectra were acquired using a hemispherical analyzer at a pass energy of 117.4 eV with a 0.4 eV energy step. Core-level spectra were recorded at a pass energy of 23.5 eV with a 0.1 eV energy step. All spectra were obtained with 90° between X-ray source and analyzer and with the use of low energy electrons and low energy argon ions for charge neutralization. After subtraction of the linear-type background, the core-level spectra were decomposed into their components with mixed Gaussian-Lorentzian (30:70) shape lines using the CasaXPS software. Quantification calculations were performed using sensitivity factors supplied by PHI.

Fourier-transform infrared spectroscopy (FTIR) was conducted on a Thermo Fisher Inc, Nicolet 8700. The nanoparticles were analyzed in the IR radiation in a pellet form, obtained by mixing 1.5 mg of lyophilized lysozyme, CuS-BSA or CuS-BSA/Lysozyme with 200 mg of potassium bromide (KBr) and grinding to enhance uniform distribution in the KBr base. The mixtures were then pressed in a hydraulic press at a pressure ~9 tons and analyzed in the FTIR range 650-4000 cm^{-1}.

Bacteria visualization. The slides were directly visualized using a Cytation™ 5 Cell Imaging Multi-Mode Reader (BioTek Instruments SAS, France) at 20× magnification with an Olympus Plan Fluorite WD 6.6 NA 0.45 objective and the corresponding excitation and emission filters (469-35/525-39 nm for GFP and 586-15/647-57 for Texas Red).

Bacteria imaging. Scanning electron microscopy (SEM) images were obtained using a Zeiss Compact Merlin instrument at 1 kV under high vacuum with a secondary electron detector. The biological samples were previously fixed with 1% glutaraldehyde solution for 30 min in the dark at room temperature, mounted on stubs and sputter-coated with a 5-nm platinum layer.

All experiments were conducted with at least duplicate or triplicate observations per condition. Data were analyzed by two-way analysis of variance (ANOVA) with Dunnett's multiple comparisons test (comparison of multiple groups) and expressed as mean ± SD. $^{*}p$ value < 0.05, $^{**}p$ value < 0.01, $^{***}p$ value < 0.001, and $^{****}p$ value < 0.0001 were considered significant compared to a control. Statistical analysis was performed with GraphPad Prism 7 (version 7.05).

Evaluation of the mechanism of apoptosis – necrosis

HeLA cells were seeded into 24-well plates at a density of 5×10^4 cells per well in 500 µL culture medium. After 24 h, the culture medium was replaced with 1 mL of various concentrations (100-200 µg mL^{-1}) of CuS-BSA/Lysozyme or control medium 4 h before irradiation. After incubation, cells were irradiated with 980 nm (1.0 W cm^{-2}) continuous mode laser for 3 min and incubated for another 20 h. Then the liquid was removed, and the cell death mechanisms was carried out using the Annexin-V and PI stainings. Briefly, the cells were directly stained with Hoechst/Annexin V-FITC/Propidium Iodide (PI) solutions. Hoechst (5 µg mL^{-1}), 1X Annexin V-FITC (Novex™ Kit, ApoDETECT™, X20 solution) and propidium iodide (1.5 µg mL^{-1}) were diluted with 1X binding buffer (10 mM HEPES/NaOH, pH 7.4, 140 mM NaCl, 2.5 mM $CaCl_2$) and the cells were incubated with 500 µL of the staining solution at room temperature for 15 min in the dark. The cells were directly visualized in 1X binding buffer using a Cytation™ 5 Cell Imaging Multi-Mode Reader (BioTek Instruments SAS, France) at 4× magnification with an Olympus Plan Fluorite WD 17 NA 0.13 objective and the corresponding excitation and emission filters (377-50/447-60 nm for Hoechst, 469-35/525-39 nm for Annexin V-FITC and 586-15/647-57

nm for propidium iodide). The addition of PI enabled viable (AnnV^{neg}/PI^{neg}), early apoptotic (AnnV^{pos}/PI^{neg}), late apoptotic (AnnV^{pos}/PI^{pos}) and necrotic (AnnV^{neg}/PI^{pos}) cells to be distinguished. Cell counting was used to quantify the number of viable, early apoptotic, late apoptotic and necrotic cells.

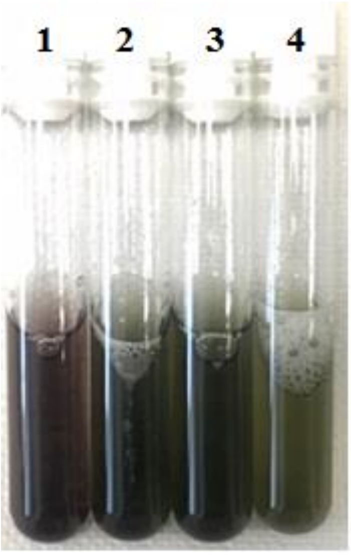

Figure S1. The long-term stability of CuS-BSA NPs at a concentration of 1 mg mL^{-1} in (**1**) DMEM medium, (**2**) DMSO, (**3**) PBS (1X, pH 7.4), and (**4**) bacterial MHB nutrient medium.

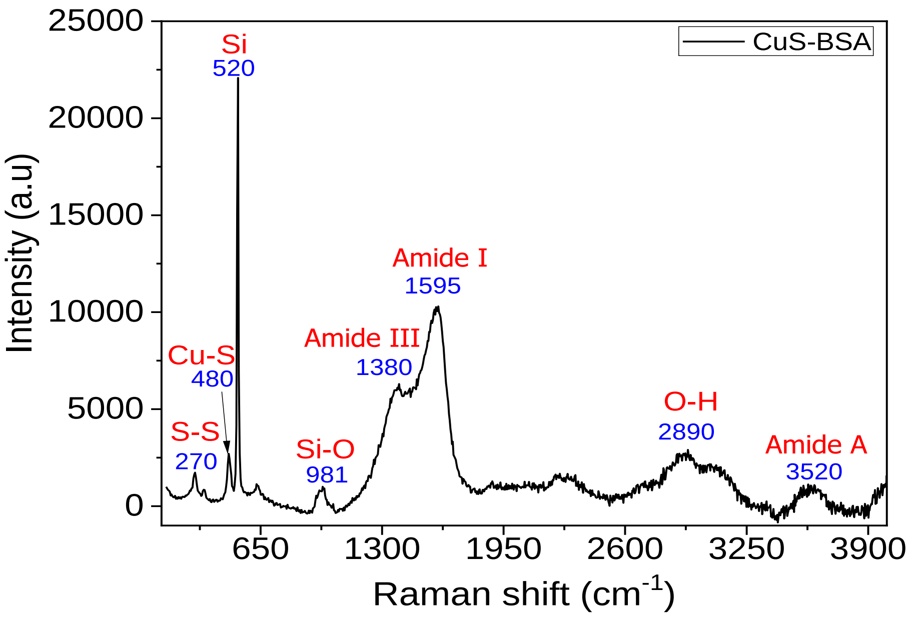

Figure S2. Raman spectrum of CuS-BSA NPs. The sharp peak at 520 cm^{-1} is due to crystalline silicon on which the CuS-BSA nanoparticles were deposited.

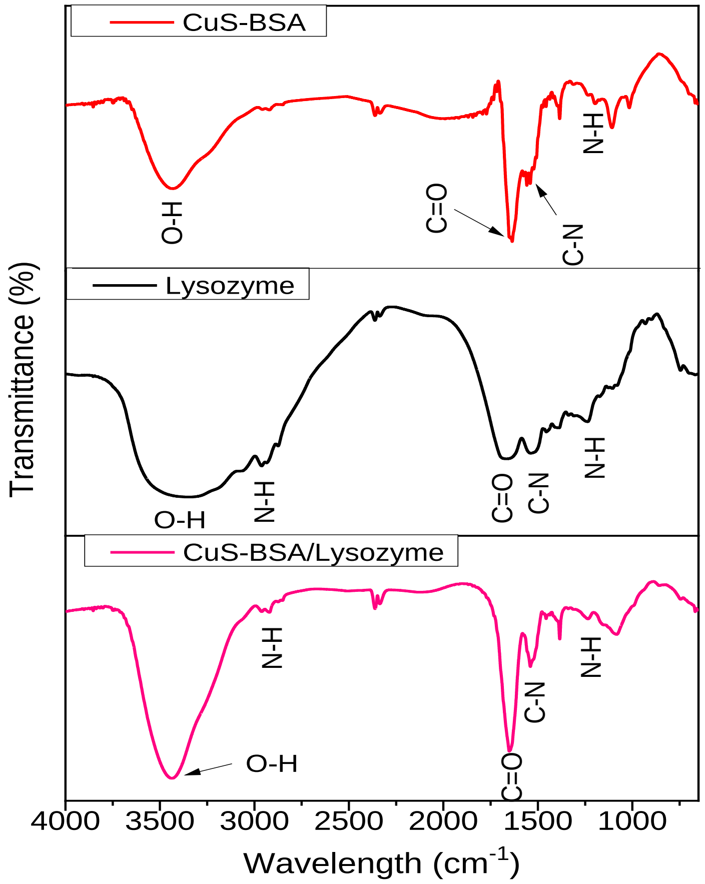

Figure S3: FTIR spectra of CuS-BSA, lysozyme and CuS-BSA/Lysozyme.

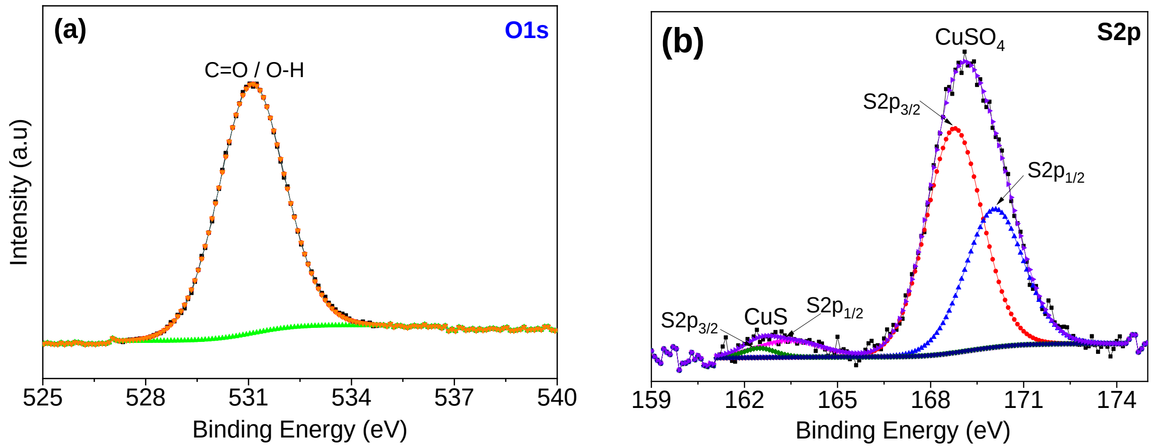

Figure S4. High-resolution XPS spectra of the O1s (**a**) and S2p (**b**) of CuS-BSA NPs.

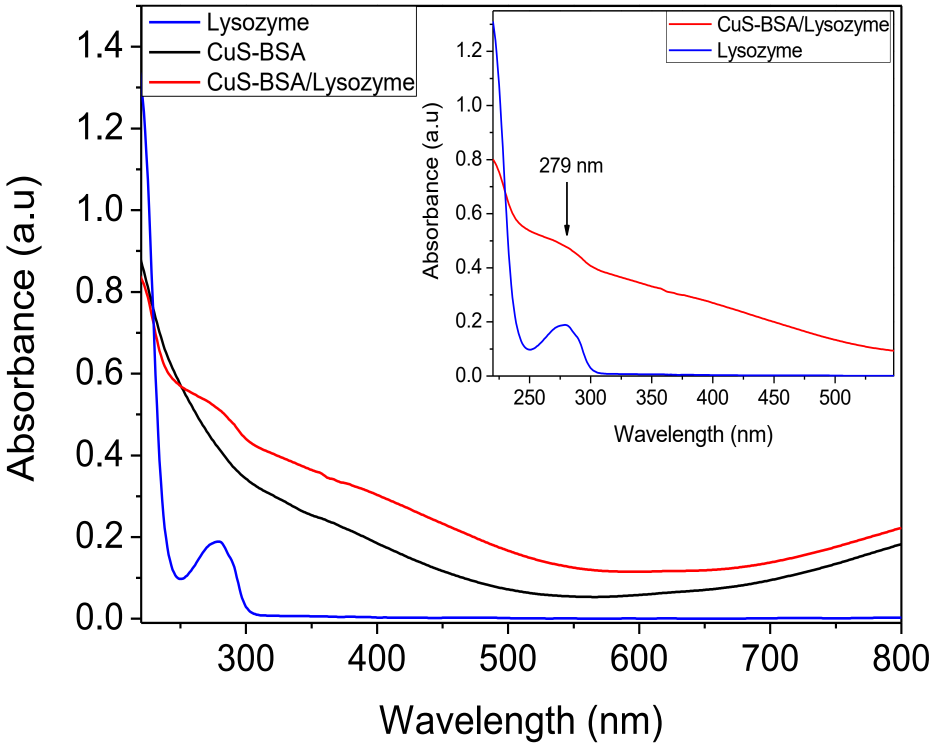

Figure S5. UV-vis absorption spectra of lysozyme (blue), CuS-BSA (black), and CuS-BSA/Lysozyme (red).

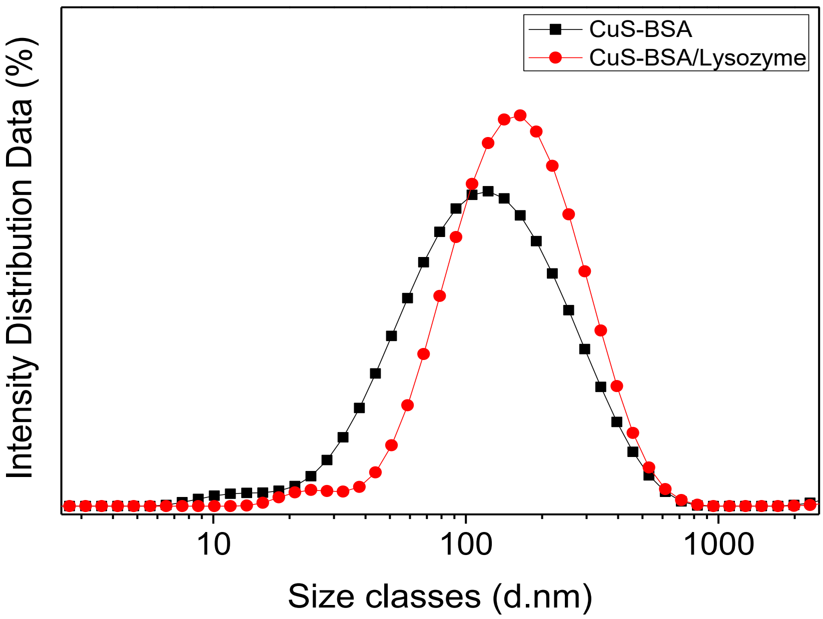

Figure S6. Dynamic light scattering (DLS) curves of CuS-BSA and CuS-BSA/Lysozyme.

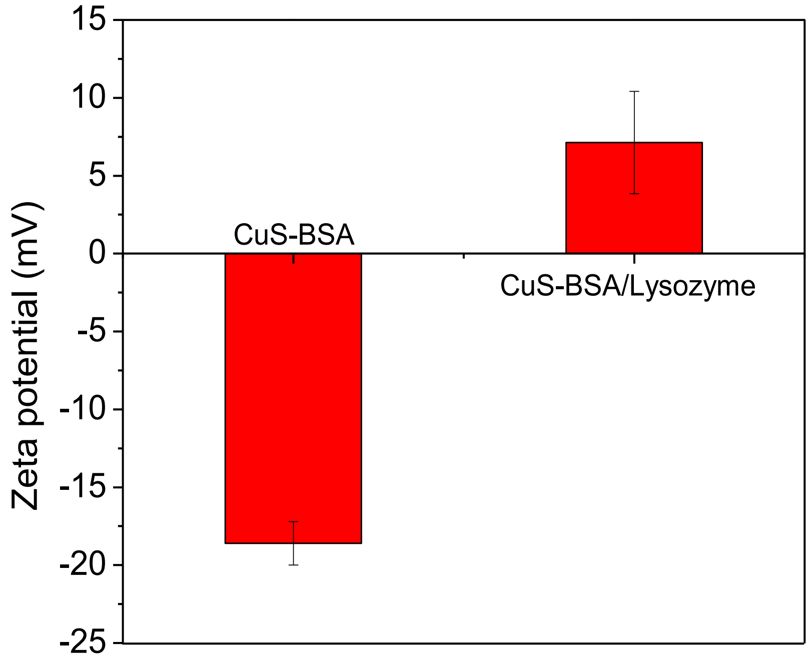

Figure S7. Comparison of zeta potential of CuS-BSA and CuS-BSA/Lysozyme in MQ-water.

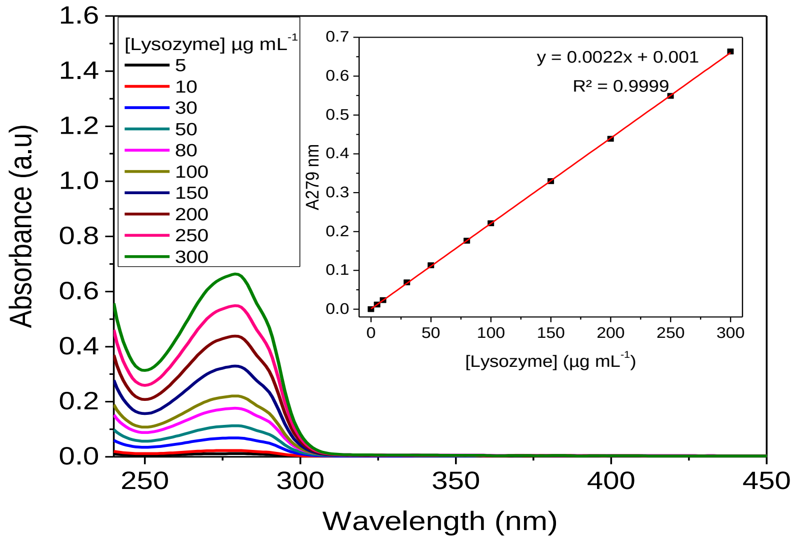

Figure S8. UV-vis absorption spectra of lysozyme in PBS (0.01 mol L^{-1}, pH 7.4) at various concentrations. Inset: The corresponding calibration curve.

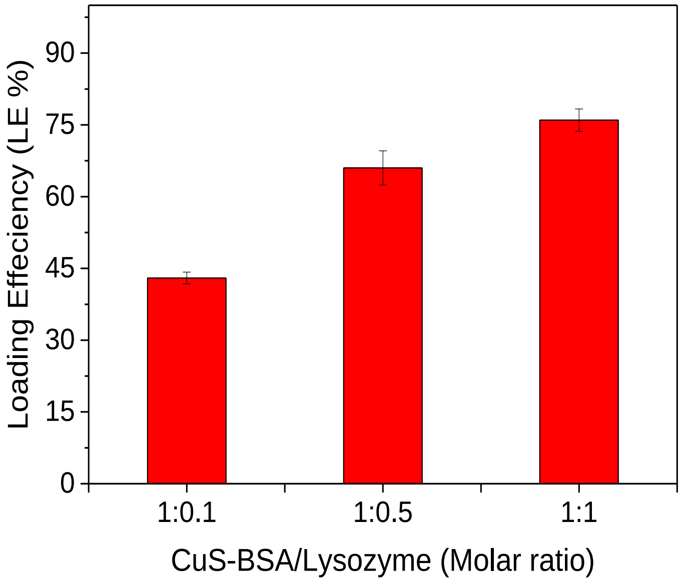

Figure S9. Lysozyme loading efficiency (LE %) by CuS-BSA in PBS (0.01 mol L^{-1}, pH 7.4).

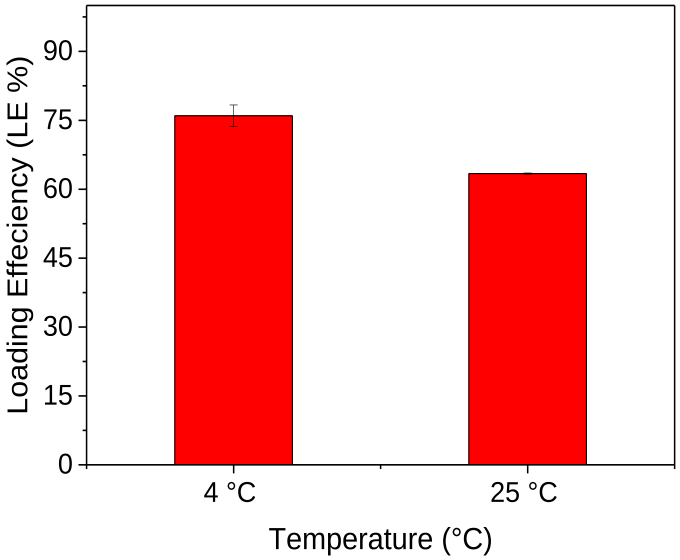

Figure S10. Lysozyme loading efficiency (LE %) onto CuS-BSA in PBS (0.01 mol L^{-1}, pH 7.4) at 4°C and 25°C.

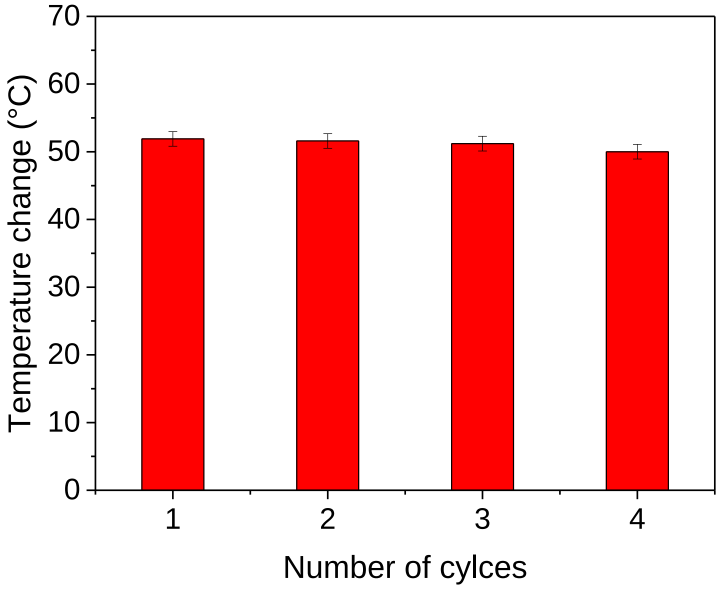

Figure S11. Temperature elevation of CuS-BSA (100 µg mL^{-1}) over four laser on/off cycles under 980-NIR laser irradiation for 3 min using a power density 0.7 W cm^{-2}.

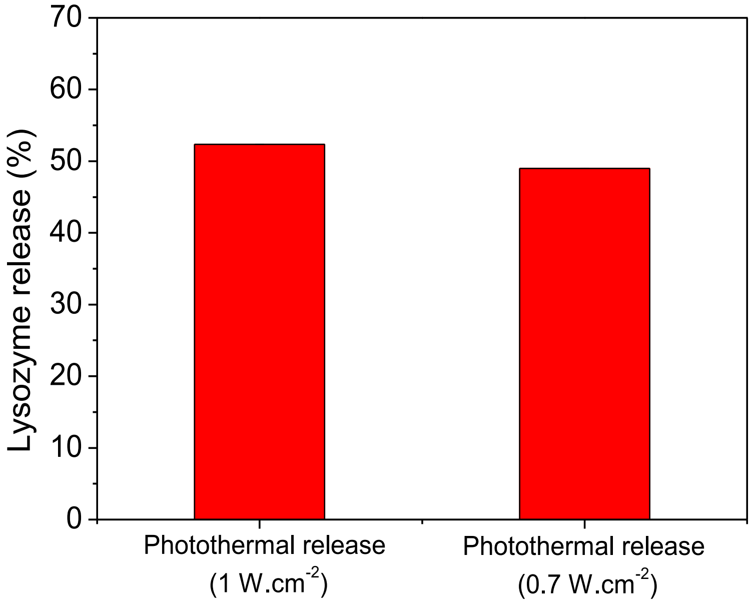

Figure S12. Photothermal release of lysozyme (%) from CuS-BSA NPs (0.1 mg mL^{-1}) after 3 min laser irradiation at 980 nm. Experiments were done in triplicate.

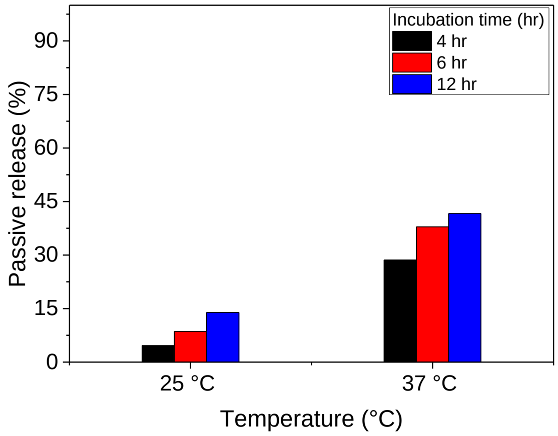

Figure S13. Passive release of lysozyme (%) from CuS-BSA NPs (0.1 mg mL^{-1}) at various temperatures and incubation times.

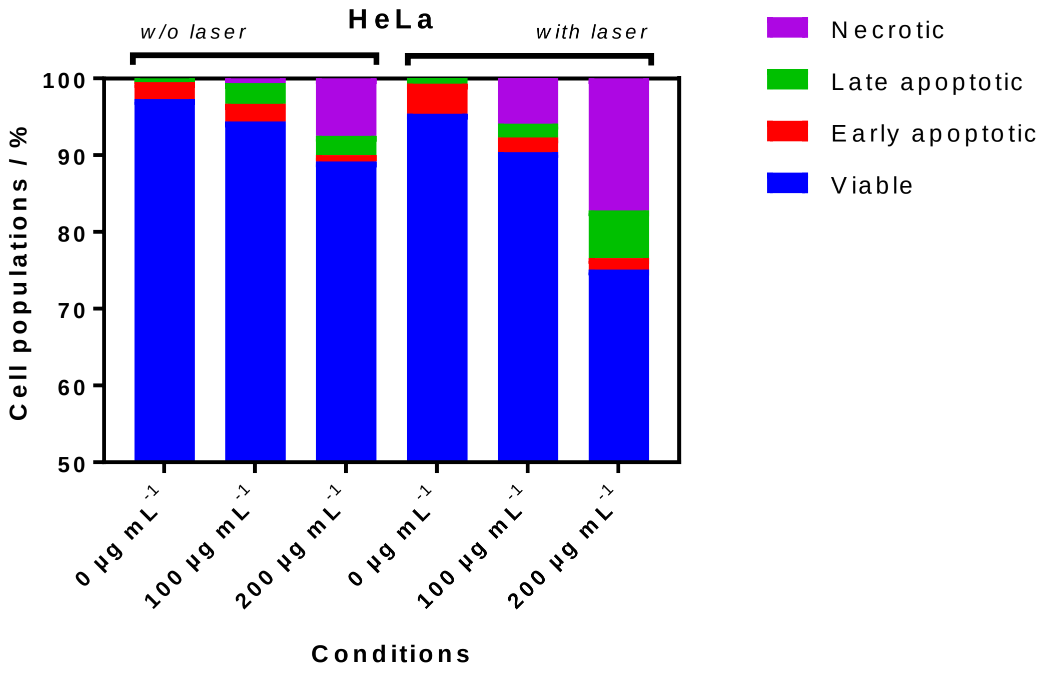

Figure S14. HeLa cell death mechanism under different conditions.

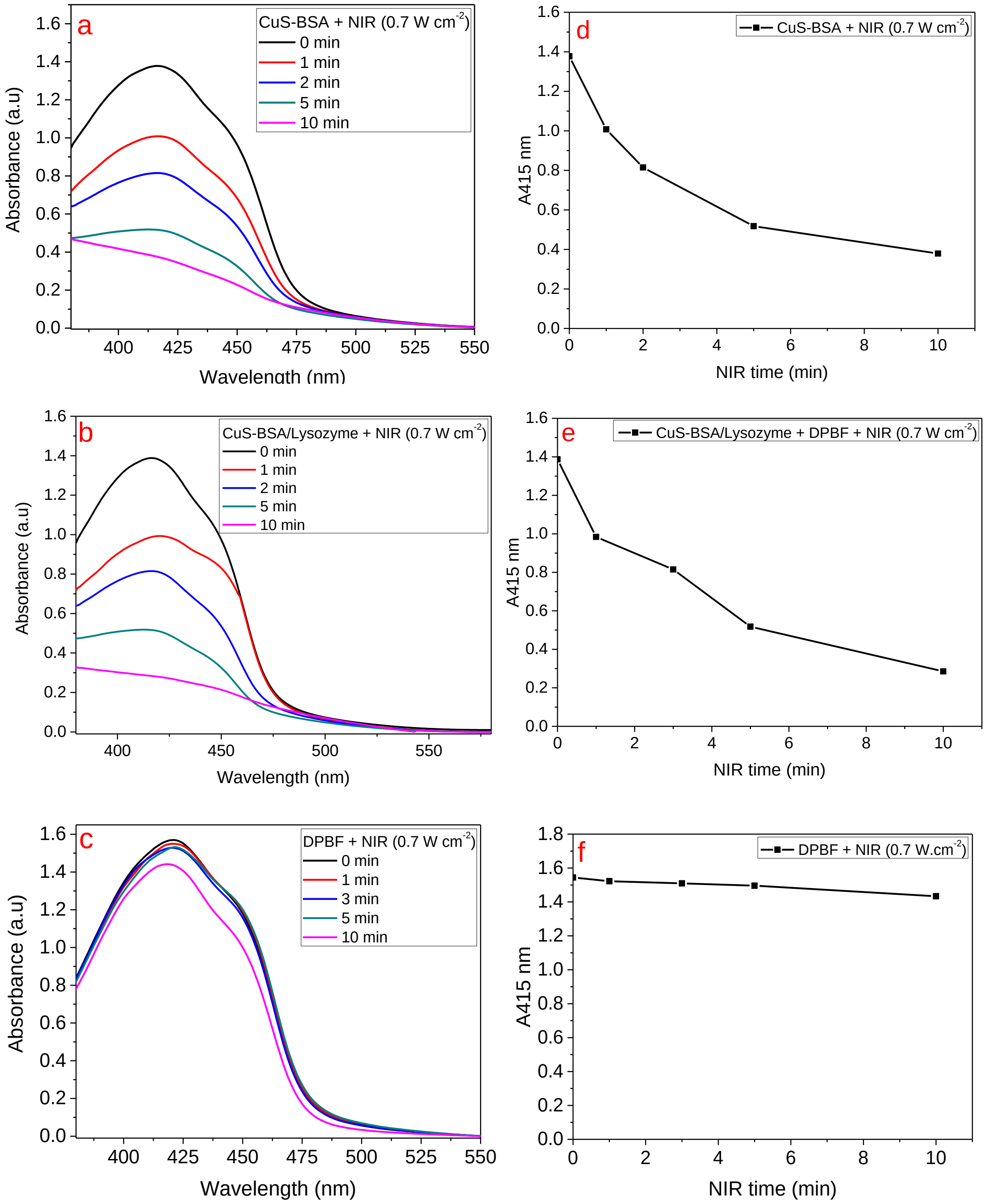

Figure S15. Time dependent DPBF quenching experiments: (**a**) CuS-BSA + DPBF + NIR (0.7 W cm^{-2}), (**b**) CuS-BSA/Lysozyme + DPBF + NIR (0.7 W cm^{-2}), (**c**) DPBF + NIR (0.7 W cm^{-2}), (**d**), (**e**) and (**f**) $^1O_2$ generation efficiency upon NIR laser irradiation.

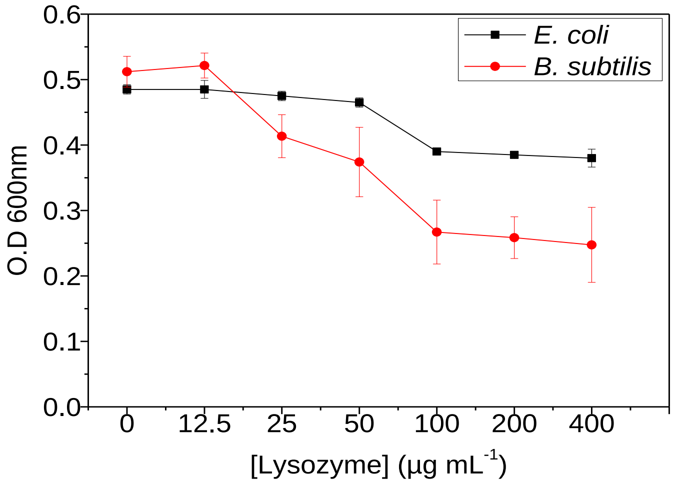

Figure S16. Change of OD_{600} in the presence of various concentrations of lysozyme (μg mL^{-1}) on *E. coli* and *B. subtilis*.

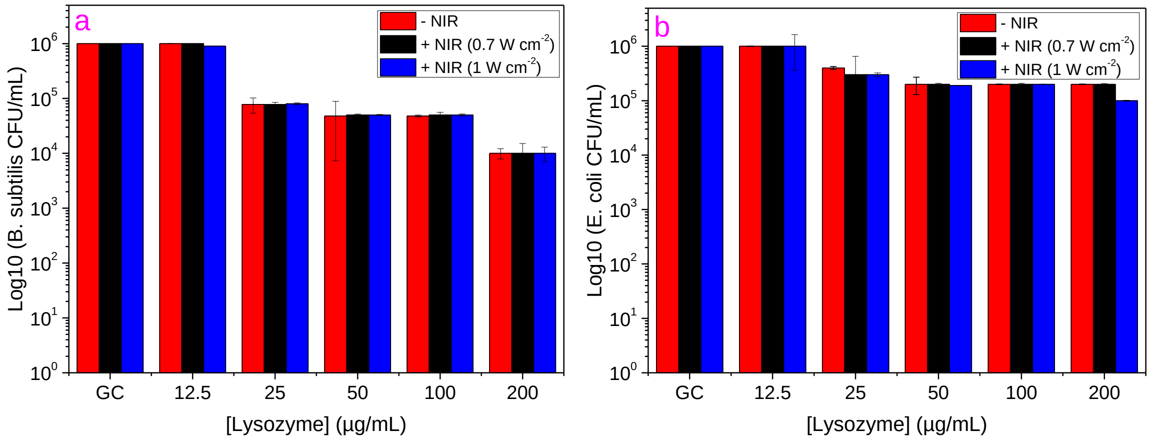

Figure S17. Antibacterial activity of lysozyme on *B. subtilis* (**a**) and *E. coli* (**b**) using various concentrations of lysozyme without and with NIR irradiation (980 nm) at different laser power densities for 3 min.
